# Road traffic noise and cardiovascular disease risk factors in UK Biobank

**DOI:** 10.1093/eurheartj/ehab121

**Published:** 2021-03-18

**Authors:** Zuzana Kupcikova, Daniela Fecht, Rema Ramakrishnan, Charlotte Clark, Yutong Samuel Cai

**Affiliations:** Acoustics, Ove Arup & Partners, 13 Fitzroy Street, London W1T 4BQ, UK; MRC Centre for Environment and Health, Department of Epidemiology and Biostatistics, School of Public Health, Imperial College London, St Mary's Campus, Norfolk Place, London W2 1PG, UK; MRC Centre for Environment and Health, Department of Epidemiology and Biostatistics, School of Public Health, Imperial College London, St Mary's Campus, Norfolk Place, London W2 1PG, UK; Nuffield Department of Women’s & Reproductive Health, Women's Centre (Level 3), John Radcliffe Hospital, University of Oxford, Oxford OX3 9DU, UK; Deep Medicine Programme, Oxford Martin School, University of Oxford, 34 Broad St, Oxford OX1 3BD, UK; Acoustics, Ove Arup & Partners, 13 Fitzroy Street, London W1T 4BQ, UK; MRC Centre for Environment and Health, Department of Epidemiology and Biostatistics, School of Public Health, Imperial College London, St Mary's Campus, Norfolk Place, London W2 1PG, UK; Nuffield Department of Women’s & Reproductive Health, Women's Centre (Level 3), John Radcliffe Hospital, University of Oxford, Oxford OX3 9DU, UK; Deep Medicine Programme, Oxford Martin School, University of Oxford, 34 Broad St, Oxford OX1 3BD, UK

**Keywords:** Transportation, Air pollution, Blood pressure, Blood lipids, Blood glucose, Inflammation

## Abstract

**Aims:**

The aim of this study was to investigate the cross-sectional associations of modelled residential road traffic noise with cardiovascular disease risk factors [systolic (SBP) and diastolic blood pressure (DBP), C-reactive protein, triglycerides, glycated haemoglobin, and self-reported hypertension] in UK Biobank.

**Methods and results:**

The UK Biobank recruited 502 651 individuals aged 40–69 years across the UK during 2006–10. Road traffic noise (*L*den and *L*night) exposure for 2009 was estimated at baseline address using a simplified version of the Common Noise Assessment Methods model. We used multivariable linear and logistic regression models, adjusting for age, sex, body mass index (BMI), smoking, alcohol intake, area- and individual-level deprivation, season of blood draw, length of time at residence, and nitrogen dioxide (main model), in an analytical sample size of over 370 000 participants. Exposure to road-traffic *L*den >65 dB[A], as compared to ≤55 dB[A], was associated with 0.77% [95% confidence interval (CI) 0.60%, 0.95%], 0.49% (95% CI 0.32%, 0.65%), 0.79% (95% CI 0.11%, 1.47%), and 0.12% (95% CI −0.04%, 0.28%) higher SBP, DBP, triglycerides, and glycated haemoglobin, respectively. Removing BMI from the main model yielded significant positive associations with all five markers with elevated percent changes. The associations with SBP or DBP did not appear to be impacted by hypertension medication while a positive association with prevalent self-reported hypertension was seen in the non-medicated group who exposed to a *L*den level of 60–65 dB[A] (odds ratio 1.07, 95% CI 1.00, 1.15).

**Conclusion:**

Exposure to road traffic noise >65 dB[A], independent of nitrogen dioxide, was associated with small but adverse changes in blood pressure and cardiovascular biochemistry.

## Graphical Abstract

**Figure:**
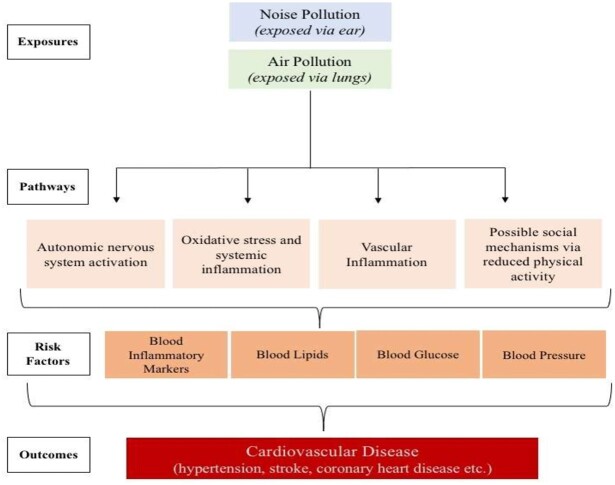
In the largest analysis to date of over 370,000 UK Biobank participants, exposure to high road traffic noise levels greater than 65dB, as compared to less than 55dB, was associated with 0.77%, 0.49%, 0.79% and 0.12% changes in SBP, DBP, triglycerides and glycated haemoglobin respectively, independent in nitrogen dioxide.

**See page 2085 for the editorial comment on this article (doi: 10.1093/eurheartj/ehab104)**

## Introduction

Road traffic noise is an important environmental risk factor for cardiovascular disease (CVD), as increasingly reported in both observational and experimental studies.^1^ However, the biological mechanisms underlying the association remain to be thoroughly elucidated. A proposed hypothesis suggests that chronic exposure to noise leads to activation of the autonomic and endocrine system, generating unfavourable changes in traditional risk factors such as blood pressure, blood lipids, and blood glucose which, if left untreated, will manifest in CVD.^2^ Long-term sleep disturbance as a result of night-time noise exposure can also impact cardiovascular health due to repeated arousal and activation of the stress cascade via autonomic and endocrine systems.^3^

Of all the cardiovascular outcomes examined to date, the relationship between road traffic noise and hypertension is the most studied. A meta-analysis by the World Health Organisation reported a 5% increase in prevalence of hypertension [95% confidence interval (CI) 2%, 8%] per 10 dB of road traffic noise based on 26 cross-sectional studies published up to 2014, for which the overall quality of evidence was rated as *very low*.^4^ Another meta-analysis of 14 cohort and case–control studies published between 2011 and 2017 reported a relative risk of 1.02 (95% CI 0.98, 1.05), for which the overall quality of evidence was rated as *low*.^5^ Both reviews indicated that further research is very likely to have an important impact on the estimated risk. Only a few studies have investigated the relationship with continuous blood pressure traits in adults and reported heterogeneous results. Some only observed a positive association with either systolic blood pressure (SBP)^6–8^ or diastolic blood pressure (DBP).^9^ One study found a null association with either measure.^10^ The pooled analysis of over 88 000 participants from three European cohorts was the largest study but reported negative associations with both measures.^11^ Studies of C-reactive protein (CRP), blood lipids and glucose are still very limited.^12–14^ Traffic-related air pollution, the impact of which on cardiovascular health is well documented,^15^ has the potential to confound associations between road traffic noise exposure and cardiovascular outcomes and, therefore, should be considered when attempting to disentangle road traffic noise effects.

Here, we examined cross-sectional associations of long-term residential road traffic noise with SBP, DBP, triglycerides, glycated haemoglobin, CRP, and self-reported hypertension, accounting for individual-level confounders including traffic-related air pollution in the largest study to date involving over 370 000 participants in UK Biobank.

## Methods

### Study population

A total of 502 651 individuals aged 40–69 years living within 25 miles of one of the 22 study assessment centres across the UK were recruited into UK Biobank during baseline assessment from 2006 to 2010.^16^ A comprehensive set of individual-level data was provided by participants using touchscreen questionnaires while biological and physical measurements were also collected. Despite a relatively low response rate (5.5%), risk factor associations in UK Biobank are likely generalizable.^17^ All participants provided written consent and ethical approval was obtained from the North West Multi-Centre Research Ethical Committee and Patient Information Advisory Group.

### Cardiovascular risk factors

Non-fasting blood samples were collected and transported in temperature-controlled boxes for storage. Serum concentrations of high-sensitivity CRP (mg/L), triglycerides (mmol/L), and glycated haemoglobin (mmol/mol) were analysed using immunoturbidimetric, glycerol phosphate oxidase peroxidase and high-performance liquid chromatography, respectively.^18^ SBP and DBP (mmHg) were measured twice using a digital Omron HEM-7015IT monitor, following a standard protocol.^19^ The mean of two measures of SBP and DBP was obtained to account for random fluctuations.

### Noise exposure assessment

Address-level annual mean road traffic noise estimates were modelled using a simplified version of the Common Noise Assessment Methods in Europe model, developed and validated for epidemiological studies.^20^ ^,^ ^21^ This simplified model has relatively good performance on exposure ranking (Spearman ratio: 0.75)^21^ and has been used in previous analyses.^14^ Annual mean A-weighted sounds pressure level in decibels (dB[A]) for 2009 was estimated based on all road sources within 500 m of residential address. The model considered detailed information on noise propagation (refraction and diffraction), absorption from buildings and land use, distance between receptor and source and angle of view, meteorology, building heights, land cover, road network geography, and calculated hourly vehicle flows using a daily average traffic profile. We used the noise indicator *L*_den_ (weighted average 24-h noise sound level, with a penalty of 5 and 10 dB added to the evening hours and night hours, respectively) and *L*_night_ (average sound pressure level during night-time hours 23:00–07:00), to be comparable to previous studies.

### Covariates

Age (in years continuous), sex (female, male), smoking status (current, past, never), alcohol intake frequency (daily or almost daily, 3–4 times a week, 1–2 times a week, occasional drinker, never), use of antihypertensive medication, and self-reported hypertension and diabetes (‘ever-had’) were obtained from questionnaires. Body mass index (BMI, kg/m^2^) was calculated using height (cm) and weight (kg) measured after removal of heavy clothes and shoes. Season of blood draw (spring, summer, autumn, winter) was recorded during clinical measurements. Time at residence of recruitment in years was obtained. Household income before tax (<£18 000, £18 000–£30 999, £31 000–51 999, £52 000–£100 000, >£100 000) and economic activity (economically active (paid employment), economically inactive (unpaid employment, unemployment, housework, retired, etc.) was used as proxies for individual socioeconomic status. Townsend deprivation index (quintiles: most deprived to least deprived) is a composite area-level indicator of material deprivation based on unemployment, non-car ownership, non-house ownership, and household overcrowding using information from the UK 2011 Census. Address-level annual average concentrations for nitrogen dioxide (NO_2_), a primary indicator of roadside air pollution, and particulate matter with a diameter of <2.5 μm (PM_2.5_), for which road vehicles are important emission sources, were integrated into UK Biobank as part of a previous study.^22^ ^,^ ^23^ Land-use regression models were used to predict annual average NO_2_ and PM_2.5_ exposure at address for year 2010. The models used AirBase routine monitoring data with geospatial variables on road network (road class, road length), land use (residential, natural, industry, urban green), population density and altitude. The model performance [explained variance (*R* ^2^) between modelled and measured exposures] was 89% and 82%, for NO_2_ and PM_2.5 _respectively.

### Statistical analysis

Descriptive analysis was conducted for covariates, exposures, and outcomes in the whole population. Spearman correlations between road traffic noise metrics and air pollutants were calculated.

The distribution of noise metric *L*_den_ was right skewed and therefore categorized as ≤55, >55 to ≤60, >60 to ≤65, and >65 dB[A] in the analysis. The reference value was set at 55 dB[A] as it is close to the median of *L*_den_ and a suggested health effect threshold set by European Union. Categorization was also applied to *L*_night_ as ≤45, >45 to ≤ 50, >50 to ≤55, and >55 dB[A]. The reference value was set at 45 dB[A] following the 2018 European noise guideline.^24^ Each risk factor was examined as a log-transformed continuous outcome to address skewness of data. CRP levels >10 mg/L were encoded as missing as levels above this value may indicate a current infection.^14^

Association between residential annual mean road traffic noise (*L*_den_ or *L*_night_) and each risk factor was analysed using multivariable linear regression. For the binary outcome of self-reported hypertension, multivariable logistic regression was used. Results for SBP, DBP, triglycerides, glycated haemoglobin and CRP were presented as percent change and 95% CI in mean differences between the reference group and other groups while results for self-reported hypertension were presented as odds ratios (ORs) and 95% CI.

The models were as follows: *Model 1*: unadjusted; *Model 2*: fully adjusted model with adjustment for age, sex, BMI, smoking status, alcohol intake frequency, household income, Townsend deprivation index, time at residence, season of blood draw, economic activity; *Model 3*: Model 2 further adjusted for NO_2_; and *Model 4*: Model 2 further adjusted for PM_2.5_. Most previous studies have accounted for NO_2_ effects; to facilitate comparisons, we set *a priori* Model 3 as the main model in our study.

Sensitivity analyses were performed to test the robustness of Model 3: (i) adjustment without BMI as BMI may be on the causal pathway; (ii) further adjustment for ever-had hypertension and diabetes to capture high-risk individuals; (iii) to address the potential issue of missing data, we used multiple imputation (*m* = 20) using chained equations with fully conditional specification of prediction equations. All covariates were included in the imputation equation; (iv) to repeat the analysis of categorical *L*_den_ by lowering the reference value to 52 dB[A], which is close to the 5th percentile of *L*_den_ distribution. The corresponding categories were ≤52, >52 to ≤55, >55 to ≤58, >58 to ≤61, >61 to ≤64 and >64 dB[A]. An increment of 3 dB was chosen as it represents a doubling of sound energy levels, which is audible to human ear as a small change in loudness.^25^

For the analyses on SBP, DBP and self-reported hypertension in Model 3, we further investigated the role of antihypertensive medication. As with a previous study,^26^ we tested different approaches: (i) to further adjust for medication; (ii) to restrict analyses to participants on medication; and (iii) to restrict analyses to non-medicated participants. To examine the assumption of linearity between *L*_den_ and SBP or DBP, we conducted restricted cubic splines analyses by placing three knots at 55, 60 and 65 dB[A] of the *L*_den_ distribution.

We explored effect modification in Model 3 *a priori* by sex, age (≥65 vs. <65 years), household income, area Townsend index, and time at current residence (≥10 vs. <10 years). All statistical analysis was performed using STATA/IC v 15.1.

## Results

The analytical sample included 502 521 participants, 54.4% were female and mean age was 56.5 years (*Table 1*). The mean *L*_den_ exposure was 56.1 dB[A], ranging from 51.5 to 93.4 dB[A]. About 12% of the study participants were exposed to a residential *L*_den_ >60 dB[A]. The mean NO_2_ and PM_2.5_ exposure levels were 26.7 and 9.9 μg/m^3^. Spearman’s correlation coefficients between *L*_den_ and NO_2_ (0.23) or PM_2.5_ (0.24) were low but were high between *L*_den_ and *L*_night_ (0.99) and between NO_2_ and PM_2.5_ (0.85).

**Table 1:** Descriptive characteristics of UK Biobank participants

General characteristics	
Total (*N*)	502 521
Sex	
Female	273 391 (54.4%)
Male	229 130 (45.6%)
Age at recruitment (years), mean ± SD	56.5 ± 8.1
Health characteristics	
BMI^a^ (*n* = 491 283)	
Underweight (<18.5 kg/m^2^)	2623 (1.0%)
Healthy (18.5–24.9 kg/m^2^)	157 409 (31.0%)
Overweight (25–29.9 kg/m^2^)	209 092 (42.6%)
Obese (>30 kg/m^2^)	122 159 (24.9%)
Smoking status (*n* = 499 571)	
Never smoker	273 527 (54.8%)
Former smoker	173 067 (34.6%)
Current smoker	52 977 (10.6%)
Alcohol intake frequency (*n* = 501 019)	
Daily or almost daily	101 774 (20.3%)
Three or four times a week	115 441 (23.0%)
Once or twice a week	129 292 (25.8%)
Occasional drinkers^b^	113 865 (22.7%)
Never drinker	40 647 (8.2%)
High blood pressure medication (*n* = 224 545)	
Yes	56 085 (25.0%)
No	168 460 (75.0%)
Socioeconomic status characteristics	
Economic status^c^ (*n* = 496 767)	
Economically active	287 162 (57.7%)
Economically inactive	209 605 (42.3%)
Townsend deprivation index at recruitment, quintiles (*n* = 501 898)	
1 (least deprived)	100 679 (20.3%)
2	100 127 (20.2%)
3	100 346 (19.2%)
4	100 372 (20.3%)
5 (most deprived)	100 374 (20.0%)
Average total household income before tax (*n* = 425 350)	
<£18 000	97 200 (22.9%)
£18 000 to £30 999	108 178 (25.4%)
£31 000 to £51 999	110 773 (26.0%)
£52 000 to £100 000	86 267 (20.3%)
>£100 000	22 932 (5.4%)
Season of blood draw^d^	
Spring	146 470 (29.2%)
Summer	131 999 (26.3%)
Autumn	119 389 (23.7%)
Winter	104 663 (20.8%)
Length of time at residence (years), mean ± SD (*n* = 499 846)	17.4 ± 12.1
Exposure characteristics	
*L*_den_, dB[A], mean ± SD, range (*n* = 495 155)	56.1 ± 4.3 (51.5–93.4)
Low (≤55 dB[A])	254 874 (51.4%)
Low–medium (>55 to ≤60 dB[A])	179 765 (36.3%)
Medium–high (>60 to ≤65 dB[A])	29 024 (5.9%)
High (>65 dB[A])	31 492 (6.4%)
*L*_night_, dB[A], mean ± SD, range (*n* = 495 155)	46.6 ± 4.3 (42.1–93.9)
Low (≤45 dB[A])	205 930 (41.6%)
Low–medium (>45 to ≤50 dB[A])	222 522 (44.9%)
Medium–high (>50 to ≤55 dB[A])	33 073 (6.7%)
High (>55 dB[A])	33 630 (6.8%)
NO_2_ (μg/m^3^), mean ± SD, range (*n* = 495 155)	26.7 ± 7.6 (12.9–108.5)
PM_2.5_ (μg/m^3^), mean ± SD, range (*n* = 461 228)	9.99 ± 1.1 (8.2–21.3)
*L*_den_, NO_2_, *r*_s_	0.23
*L*_den_, PM_2.5_, *r*_s_	0.24
Outcome characteristics,^e^	
Systolic blood pressure (mmHg), mean ± SD, range (*n* = 456 977)	137.8 ± 18.6, (65.0–253.5)
Diastolic blood pressure (mmHg), mean ± SD, range (*n* = 456 989)	82.2 ±10.1 (36.5–120.0)
C-reactive protein (mg/L), median ± IQR, range (*n* = 449 139)	1.3 ± 1.9 (0.1–10.0)
Triglycerides (mmol/L), median ± IQR, range (*n* = 469 226)	1.8 ± 1.0 (0.2–11.3)
Glycated haemoglobin (mmol/mol), mean ± SD, range (*n* = 455 865)	35.4 ± 4.3 (15.0–54.0)
Self-reported hypertension (*n* = 500 298)	
Yes	135 539 (27.1%)
No	364 539 (72.9%)

BMI, body mass index; IQR, interquartile range; NO_2_, nitrogen dioxide; PM_2.5_, particulate matter with a diameter <2.5 μm; SD, standard deviation; WHO, World Health Organisation.

a BMI categorized according to WHO and UK classifications.

b Includes individuals who drink on special occasions only and individuals who drink ∼1–2 times a month.

c Economic status refers to paid employment and unpaid employment.

d Based on date attending assessment centre: winter (December, January, February), spring (March, April, May), summer (June, July August), autumn (September, October, November).

e Median ± IQR used where mean differed from median by >10%.

**Figure 1:**
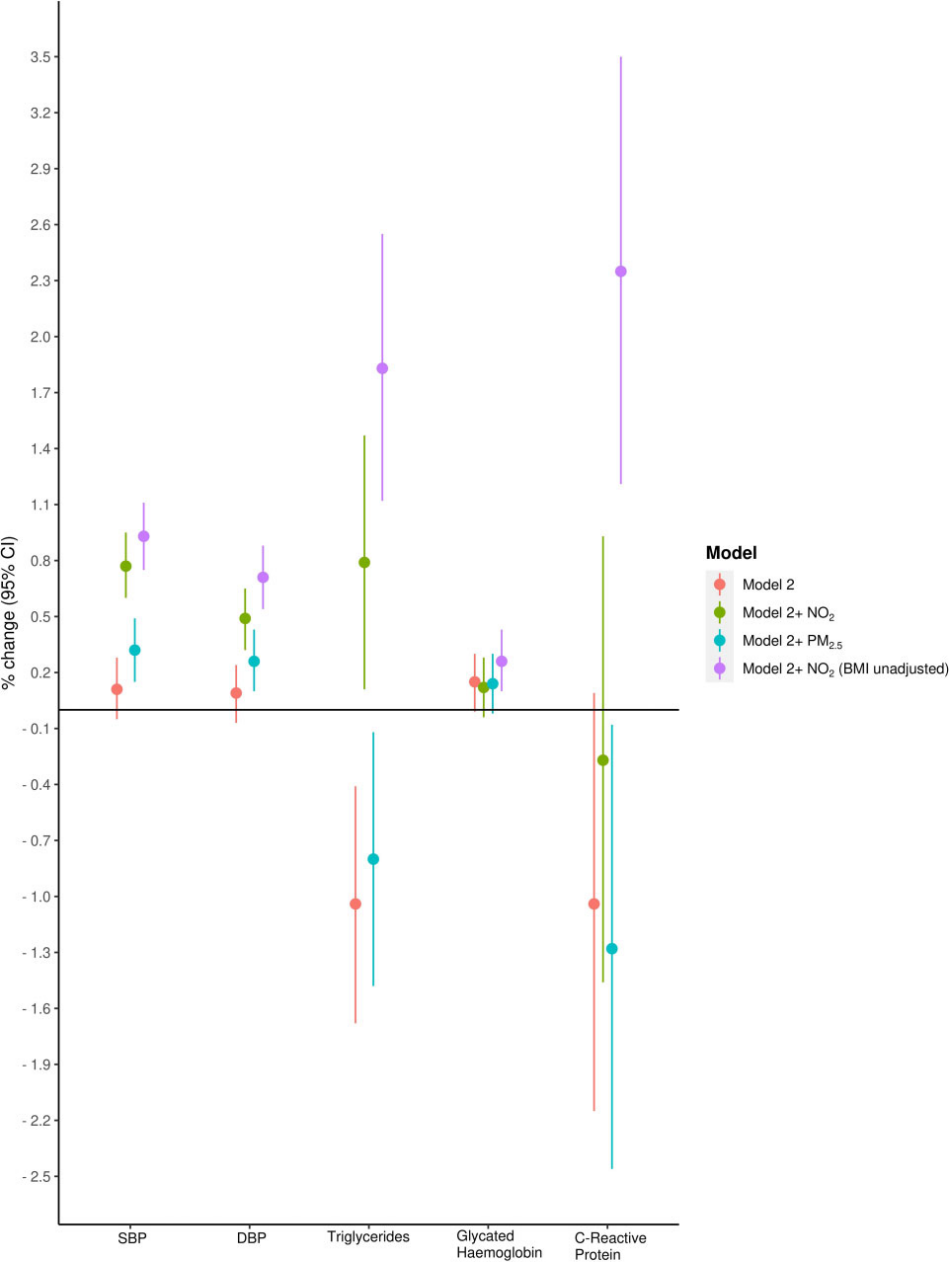
A summary of cross-sectional associations between road-*L*_den_ and percent changes in cardiovascular risk factors, comparing >65 to ≤55 dB. Model 2: fully adjusted model. Adjusted for sex, age, BMI, smoking status, alcohol intake frequency, Townsend deprivation index, household income, economic status, season of blood draw, length of time at residence.

**Figure 2:**
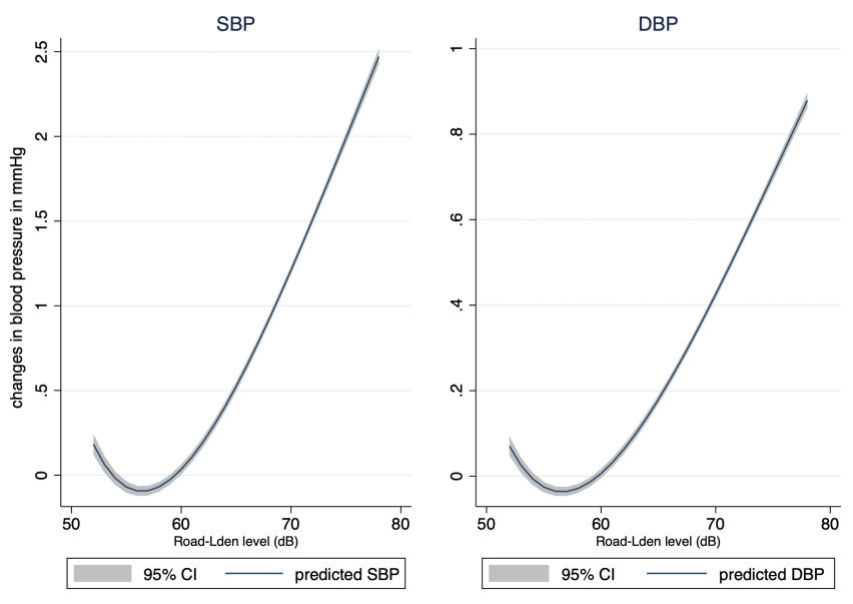
Cross-sectional changes (mmHg) in systolic and diastolic blood pressures in relation to road traffic noise exposure in Model 3 (fully adjusted model + NO_2_) based on the restricted cubic spline analysis. Model 3: adjusted for sex, age, BMI, smoking status, alcohol intake frequency, Townsend deprivation index, household income, economic status, season of blood draw, length of time at residence, and nitrogen dioxide.

In the fully adjusted Model 2, exposure to road-*L*_den_ >65 dB[A], as compared to those exposed ≤55 dB[A], was positively associated with SBP [0.11% (95% CI −0.05, 0.28)]; after further adjustment for NO_2_ or PM_2.5_ exposure, the effect estimate changed to 0.77% (95% CI 0.60, 0.95) (Model 3) and 0.32% (95% CI 0.15, 0.49) (Model 4), respectively (*Table 2* and *Figure 1*). Similarly, for DBP, after further adjustment for NO_2_ or PM_2.5_, the effect estimate changed from 0.09% (95% CI −0.07, 0.24) to 0.49% (95% CI 0.32, 0.65) and 0.26% (95% CI 0.10, 0.43), respectively. The restricted cubic spline analysis showed that the relationship between road-*L*_den_ and SBP or DBP only seems to be linear at levels >60 dB (*Figure 2*). No positive association between road-*L*_den_ and prevalence of self-reported hypertension was observed in the adjusted models (*Table 2*).

**Table 2:** The association between exposure to road traffic noise (*L*_den_) and cardiovascular disease risk factors

Noise exposure (*L*_den_)	*N*	Model 1, % change (95% CI)	*N*	Model 2, % change (95% CI)	*N*	Model 3, % change (95% CI)	*N*	Model 4, % change (95% CI)
Systolic blood pressure (mmHg)	451 132		378 302		378 302		365 675	
Low (≤55 dB[A])		Reference		Reference		Reference		Reference
Low–medium (>55 to ≤60 dB[A])		**−0.31 (−0.40, −0.23)**		**−0.10 (−0.19, −0.02)**		−−0.04 (−0.12, 0.05)		−−0.06 (−0.15, 0.03)
Medium–high (>60 to ≤65 dB[A])		**−0.53 (−0.70, −0.36)**		−0.11 (−0.28, 0.05)		−−0.03 (−0.20, 0.13)		−−0.04 (−0.21, 0.13)
High (>65 dB[A])		**−0.25 (−0.41, −0.09)**		0.11 (−0.05, 0.28)		**0.77 (0.60, 0.95)**		**0.32 (0.15, 0.49)**
Diastolic blood pressure (mmHg)	450 843		378 073		378 073		365 454	
Low (≤55 dB[A])		Reference		Reference		Reference		Reference
Low–medium (>55 to ≤60 dB[A])		−−0.04 (−0.12, 0.04)		−0.06 (−0.14, 0.02)		−−0.02 (−0.10, 0.06)		0.04 (−0.12, 0.05)
Medium–high (>60 to ≤65 dB[A])		**−0.24 (−0.40, −0.09)**		−0.16 (−0.32, 0.00)		−−0.11 (−0.27, 0.05)		−−0.11 (−0.27, 0.05)
High (>65 dB[A])		0.11 (−−0.04, 0.27)		0.09 (−0.07, 0.24)		**0.49 (0.32, 0.65)**		**0.26 (0.10, 0.43)**
Triglycerides (mmol/L)	462 342		389 392		389 392		361 072	
Low (≤55 dB[A])		Reference		Reference		Reference		Reference
Low–medium (>55 to ≤60 dB[A])		−−0.29 (−−0.62, 0.03)		−0.28 (−0.61, 0.04)		−−0.10 (−0.43, 0.22)		−−0.16 (−0.50, 0.18)
Medium–high (>60 to ≤65 dB[A])		**−0.89 (−1.53, −0.24)**		**−0.79 (−1.43, −0.14)**		−−0.57 (−1.22, 0.08)		**−0.81 (−1.48, −0.14)**
High (>65 dB[A])		−−0.31 (−−0.94, 0.32)		**−1.04 (−1.68, −0.41)**		**0.79 (0.11, 1.47)**		**−0.80 (−1.48, −0.12)**
Glycated haemoglobin (mmol/mol)	449 135		380 510		378 890		351 368	
Low (≤55 dB[A])		Reference		Reference		Reference		Reference
Low–medium (>55 to ≤60 dB[A])		0.03 (−−0.04, 0.11)		0.05 (−0.03, 0.13)		0.05 (−0.03, 0.13)		0.06 (−0.02, 0.14)
Medium–high (>60 to ≤65 dB[A])		−−0.08 (−−0.23, 0.08)		−0.04 (−0.20, 0.11)		−−0.05 (−0.20, 0.11)		−−0.10 (−0.26, 0.06)
High (>65 dB[A])		**0.36 (0.21, 0.51)**		0.15 (−0.01, 0.30)		0.12 (−0.04, 0.28)		0.14 (−0.02, 0.30)
C-reactive protein (mg/L)	442 544		373 261		373 261		346 188	
Low (≤55 dB[A])		Reference		Reference		Reference		Reference
Low–medium (>55 to ≤60 dB[A])		0.27 (−−0.34, 0.87)		−0.44 (−1.02, 0.14)		−−0.36 (−0.94, 0.22)		−−0.60 (−1.20, 0.01)
Medium–high (>60 to ≤65 dB[A])		−0.48 (−−1.68, 0.74)		−1.12 (−2.26, 0.04)		−−1.03 (−2.18, 0.13)		−−1.17 (−2.36, 0.03)
High (>65 dB[A])		**1.68 (0.50, 2.88)**		−1.04 (−2.15, 0.09)		−−0.27 (−1.46, 0.93)		**−1.28 (−2.46, −0.08)**

Noise exposure (*L*_den)_	*N*	Model 1, odds ratio (95% CI)	*N*	Model 2, odds ratio (95% CI)	*N*	Model 3, odds ratio (95% CI)	*N*	Model 4, odds ratio (95% CI)

Self-reported hypertension	492 993		413 845		413 845		384 204	
Low (≤55 dB[A])		Reference		Reference		Reference		Reference
Low–medium (>55 to ≤60 dB[A])		1.00 (0.99, 1.01)		1.00 (0.98, 1.01)		0.99 (0.98, 1.01)		0.99 (0.97, 1.00)
Medium–high (>60 to ≤65 dB[A])		1.00 (0.97, 1.03**)**		0.98 (0.95, 1.02)		0.98 (0.95, 1.01)		0.97 (0.94, 1.00)
High (>65 dB[A])		1.03 (1.00, 1.05)		0.99 (0.96, 1.02)		**0. 95 (0.92, 0.98)**		0.97 (0.94, 1.00)
Continuous *L*_den_, per 1 dB[A]		1.00 (1.00, 1.00)		1.00 (1.00, 1.00)		1.00 (0.99, 1.00)		1.00 (0.99, 1.00)

Model 1: unadjusted crude model. Model 2: fully adjusted model. Adjusted for sex, age, BMI, smoking status, alcohol intake frequency, Townsend deprivation index, household income, economic status, season of blood draw, length of time at residence. Model 3: fully adjusted model (Model 2) + adjusted for NO_2_. Model 4: fully adjusted model (Model 2) + adjusted for PM_2.5_. Bold represents significance at *P* < 0.05.

BMI, body mass index; CI, confidence interval.

The significant positive associations with both SBP and DBP found in the highest noise group in Model 3 did not appear to be affected by further adjusting for antihypertensive medication use or restricting analyses to those with or without medication (*Table 3*). In contrast, higher positive ORs were observed for self-reported hypertension among the non-medicated participants who exposed to road-*L*_den_ of 55–60 dB (1.03, 95% CI 0.99, 1.06) and of 60–65 dB (1.07, 95% CI 1.00, 1.15), but the association was null in the highest noise group.

**Table 3:** The association between road traffic noise and systolic blood pressure, diastolic blood pressure, and self-reported hypertension, stratified by high blood pressure medication intake

Noise exposure, *L*_den_	*N*	Model 3—further adjusted for antihypertensive medication, % change (95% CI)	*N*	Model 3—restricted to subjects with antihypertensive medication, % change (95% CI)	*N*	Model 3—restricted to subjects without antihypertensive medication, % change (95% CI)	*P*_Interaction*_
Systolic blood pressure (mmHg)	178 409		43 384		135 025		
Low (≤55 dB[A])		Reference		Reference		Reference	
Low–medium (>55 to ≤60 dB[A])		0.09 (−0.02, 0.21)		0.16 (−0.08, 0.41)		0.09 (−0.04, 0.22)	**0.00 (+)**
Medium–high (>60 to ≤65 dB[A])		**0.25 (0.02, 0.15)**		**0.60 (0.11, 1.11)**		0.13 (−0.13, 0.39)	**0.00 (+)**
High (>65 dB[A])		**0.75 (0.51, 0.99)**		**0.75 (0.23, 1.26)**		**0.73 (0.47, 1.00)**	**0.00 (+)**
Diastolic blood pressure (mmHg)	178 238		43 346		134 892		
Low (≤55 dB[A])		Reference		Reference		Reference	
Low–medium (>55 to ≤60 dB[A])		0.00 (−0.11, 0.11)		0.03 (−0.21, 0.27)		0.01 (−0.12, 0.15)	0.10 (−)
Medium–high (>60 to ≤65 dB[A])		−0.02 (−0.25, 0.20)		0.00 (−0.59, 0.49)		−0.06 (−0.31, 0.20)	0.87 (−)
High (>65 dB[A])		**0.51 (0.27, 0.75)**		**0.64 (0.14, 1.16)**		**0.44 (0.17, 0.71)**	0.64 (−)

Noise exposure, *L*_den_		Model 3—further adjusted for antihypertensive medication, odds ratio (95% CI)		Model 3—restricted to subjects with antihypertensive medication, odds ratio (95% CI)		Model 3—restricted to subjects without antihypertensive medication, odds ratio (95% CI)	*P*_Interaction_

Self-reported hypertension	193 911		47 284		146 625		
Low (≤55 dB[A])		Reference		Reference		Reference	
Low–medium (>55 to ≤60 dB[A])		1.02 (0.98, 1.05)		0.99 (0.92, 1.05)		1.03 (0.99, 1.06)	**0.00 (+)**
Medium–high (>60 to ≤65 dB[A])		1.06 (0.99, 1.13)		1.00 (0.88, 1.14)		1.07 (1.00, 1.15)	**0.00 (+)**
High (>65 dB[A])		0.97 (0.91, 1.04)		0.89 (0.78, 1.02)		0.99 (0.93, 1.07)	**0.00 (+)**
Continuous *L*_den_, per 1 dB[A]		1.00 (1.00, 1.00)		0.99 (0.99, 1.00)		1.00 (1.00, 1.00)	**0.00 (+)**

Model 3: fully adjusted model (Model 2) + adjusted for NO_2_. *P*_interaction*_: interaction between road traffic noise (*L*_den_) and high blood pressure medication; sign in the bracket indicates the direction of the interaction term. Bold represents significance at *P* < 0.05.

CI, confidence interval.

In the fully adjusted Model 2, exposure to road-*L*_den_ >65 dB[A], as opposed to those exposed ≤55 dB[A], was negatively associated with triglycerides [−1.04% (95% CI −1.68, −0.41)]; after further adjustment for NO_2_ exposure, there was a significant positive association [0.79% (95% CI 0.11, 1.47)] (*Table 2* and *Figure 1*). However, when PM_2.5_ was further adjusted, the direction and strength of the association was opposite to those in which adjustment of NO_2_ was made.

Comparing road-*L*_den_ >65 vs. ≤55 dB[A], a positive association was found with glycated haemoglobin [0.15% (95% CI −0.01, 0.30)] in Model 2; after further adjustment for either air pollutant did not change the effect estimate materially (*Table 2* and *Figure 1*). Associations with CRP were largely negative and non-significant, except in the model when PM_2.5_ was further considered [−1.28% (95% CI −2.46, −0.08)].

Similar significant positive associations were observed for SBP, DBP and triglycerides when comparing *L*_night_ >55 vs. ≤ 45 dB[A] in the models further adjusted for NO_2_ (Supplementary material online, *Appendix SA*).

Sensitivity analyses on our main model, Model 3 (Model 2 further adjusted for NO_2_), by not adjusting for BMI, altered the results (*Figure 1* and Supplementary material online, *Appendix SB*). The effect estimates for the significant positive associations between *L*_den_ and SBP, DBP, and triglycerides in the highest exposure group (>65 dB) increased by 20%, 45%, and 131%, respectively, while significant positive associations were also seen for glycated haemoglobin [0.26% (95% CI 0.10, 0.43)] and CRP [2.35% (95% CI 1.21, 3.50)]. Effect estimates did not change materially by further adjusting for ever-had hypertension or diabetes in Model 3 (Supplementary material online, *Appendix SC*). Significant positive associations were still observed for SBP, DBP, and triglycerides in the >65 dB group based on the analyses of the imputed datasets in Model 3 (Supplementary material online, *Appendix SD*), with similar effect estimates as those in *Table 2*. Results also remained similar when the reference value was lowered to *L*_den_ to ≤52 dB[A] in the analyses (Supplementary material online, *Appendix SE*).

Effect modification by sex was observed for SBP, glycated haemoglobin, and CRP (Supplementary material online, *Table SF1*) and by household income was observed for SBP and DBP (Supplementary material online, *Table SF2*). In the highest noise group >65 dB[A], positive associations with higher estimates were seen for CRP for females and glycated haemoglobin for males, but neither were statistically significant (Supplementary material online, *Table SF3*). Individuals aged ≥65 years, exposed to road-*L*_den_ of 55–60 dB[A], had a higher estimated change (0.13%) in haemoglobin as compared to that (−0.01%) of individuals aged <65 years (Supplementary material online, *Table SF4*). A significant positive association (0.21%, 95% CI 0.03, 0.39) was found with haemoglobin in the highest noise group among individuals aged <65 years. No effect modification by either time at residence or area Townsend index was observed (Supplementary material online, *Table SF5*). Stronger associations with both SBP and DBP were seen in the higher household income groups comparing >65 vs. ≤ 55 dB[A] (Supplementary material online, *Table SF6*).

## Discussion

In the UK Biobank, comparing annual average residential road traffic *L*_den_ >65 vs. ≤55 dB[A], the positive estimates of SBP and DBP became significantly higher after further adjusting for either NO_2_ or PM_2.5_. Current use of antihypertensive medication did not appear to affect these associations. The association with glycated haemoglobin was positive and not confounded by either pollutant; in contrast, the associations with both triglycerides and CRP were less stable, depending on whether NO_2_ or PM_2.5_ was further adjusted. Another key finding suggests that BMI may be on the causal pathway between road traffic noise and these traditional CVD markers as excluding BMI from the analyses yielded significant positive associations in the highest noise group (>65 dB[A]) among all five markers.

### Blood pressure and hypertension

To date, only a handful of studies have investigated the association between long-term road traffic noise exposure and changes in blood pressure among adults. The estimated associations across these previous studies are not consistent. Three studies from Spain,^7^ Switzerland,^8^ and Denmark^6^ reported a significant positive association with SBP but not DBP, and only among subgroups (men, older participants, or diabetic individuals),^6^ ^,^ ^8^ or when night-time indoor bedroom noise exposure (i.e. with reduced exposure misclassification) was used in the analysis.^7^ In contrast, a German study only observed a significant positive association with DBP but not SBP, and the association was stronger among men or diabetic individuals.^9^ A London study analysed night-time road traffic noise at both continuous and categorical scales but found no association with either blood pressure measure.^10^ These studies had a sample size ranging from 2500 to 44 000 participants. A pooled analysis of individual-level data from three European cohorts (Lifelines, HUNT3, and EPIC-Oxford) was the largest previous study with a sample size of over 88 000 participants.^11^ Unexpectedly, this study reported a significant negative association with DBP, which may be mainly driven by the Lifelines cohort, the largest and youngest cohort (mean age of 44 years) among the three. When categorical noise levels and blood pressure associations were analysed by cohort, significant positive associations were found in the >60 dB group vs. ≤50 dB, for both SBP and DBP in HUNT3, while in the EPIC-Oxford cohort, the association with SBP was positive in the highest >65 dB group vs. ≤55 dB. These findings, however, should be interpreted with caution as the number of participants exposed to the highest noise group in each cohort was relatively small (*N* = 230 for HUNT3 and *N* = 580 for EPIC-Oxford). Most of these previous studies analysed the relationship assuming a linear relationship and some found that the relationship was not confounded by air pollution^7^ ^,^ ^11^ by analysing models without and with air pollution adjustment.

Based on the largest study sample to date, in which over 30 000 participants were classified as highly exposed to residential road traffic noise (i.e. >65 dB), our study offers some new insights into this relationship. First, we found significant positive associations with both SBP and DBP only among highly exposed participants, suggesting that the relationship is likely non-linear. The restricted cubic spline analyses further revealed that the relationship only seems to be linear at levels above 60 dB. Second, the positive effect estimates were significantly amplified upon inclusion of either NO_2_ or PM_2.5_ into the model, suggesting that the effect estimates of road traffic noise on both SBP and DBP may be underestimated without accounting for air pollution effect, particularly that from near-road traffic as indicated by NO_2_ in our analysis. It may be possible that NO_2_ mainly originates from road traffic while PM_2.5_ could have many sources other than road traffic, thereby contributing in part to a stronger confounding effect by NO_2_. NO_2_ could also be a proxy of ultrafine particles, a pollutant sharing very similar propagation behaviour to noise^27^ and was recently linked to short-term adverse changes in blood pressure.^28^ As recently pointed out, it is still not completely clear whether traffic noise and air pollution have differing, additive, synergistic and/or antagonistic effects on cardiovascular outcomes.^2^ Third, we tested different approaches to accounting for antihypertensive medication use but the results remained robust.

The overall quality of evidence on the association between road traffic noise and prevalent or incident hypertension is rated as *very low* to *low*.^4^ ^,^ ^5^ We observed a null cross-sectional association with self-reported hypertension in the UK Biobank cohort. This is consistent with two recent London-based studies on both prevalent or incident hypertension.^10^ ^,^ ^29^ The ESCAPE study found that the pooled positive estimate, from six European cohorts, on incident self-reported hypertension was attenuated to null after adjusting for PM_2.5_.^30^ More recently, a Danish study reported no association between road traffic noise exposure and prescriptions for hypertension medication after a 14-year follow-up^31^ while a Ontario study found a significant 2% increase in incident hypertension with a 15-year follow-up.^32^ The latter study was based on a health insurance database covering over 700 000 participants with the assignment of noise estimates at postcode level and a lack of adjustment for lifestyle factors. Despite we found a null association among all participants, our analyses did show that the associations with self-reported hypertension tended to be positive and stronger among those who were currently not using any antihypertensive medication and exposed to an *L*_den_ level between 55 and 65 dB. This finding suggests that long-term exposure to moderate/high levels of road traffic noise may be harmful on potentially uncontrolled hypertension.

### Glycated haemoglobin, triglycerides, and C-reactive protein

The positive associations with glycated haemoglobin were consistent across all adjusted models, without or with air pollution adjustment. In a recent analysis of the Lifelines cohort, a significant positive association was found with blood glucose but not glycated haemoglobin, independent of air pollution adjustment.^14^ While studies of road traffic noise on blood glucose levels remain scarce, studies evaluating associations with diabetes generally reported an increased risk, for example a meta-analysis showed a 7% (95% CI 2%, 12%) increased risk for every 5 dB higher road traffic noise exposure.^33^

We found opposite associations with triglycerides upon adjustment of NO_2_ or PM_2.5_. Two studies, of young^34^ or middle-aged^35^ adults, found that the estimated changes in triglycerides of long-term exposure to NO_2_ were much higher as compared to PM_2.5_ while studies on long-term traffic noise exposure remain few. In our previous work, the positive association with triglycerides no longer remained significant after adjustment for NO_2_ or PM_10_ in the cohorts of Lifelines and HUNT3.^14^ The relative importance of gaseous and particulate pollutants on blood lipid profiles, and their respective interactions with traffic noise, is unclear. Future experimental and epidemiological studies are warranted to investigate both traffic noise and air pollution on lipid metabolism.

In a previous analysis of the Lifelines and HUNT3 cohorts, a positive association between long-term road traffic noise exposure and CRP was observed; however, the estimate was slightly attenuated but remained positive after controlling for air pollution.^14^ This is in contrast with our findings of negative associations in air pollution-adjusted models. The inconsistency between the two studies may be because our model had also adjusted for both BMI and area-level socioeconomic status. Recently, the population-based SAPALDIA cohort in Switzerland found significant enrichment of DNA methylation relating to CRP, independent of other noise sources and air pollution, with road *L*_den_ exposure.^36^ This is in line with findings from novel experimental models, which found that traffic noise could induce oxidative stress and inflammation in the blood and the vasculature via an increased level of angiotensin II.^1^ More evidence is needed to support this novel mechanism through systemic inflammation for the association between traffic noise and health outcomes.

### Role of body mass index

Notably, we observed stronger significant positive associations for all five risk factors after un-adjusting for BMI. This raises the possibility that BMI may be on the causal pathway on the investigated associations between traffic noise and cardiovascular health. A handful of studies in Europe, including the UK Biobank study, have suggested a positive association between long-term exposure to traffic noise and adiposity markers.^37^ We suspect that BMI may likely serve as a mediator rather than a confounder in our investigated associations and this speculation should necessitate a formal mediation analysis in future works. In particular, exploring the potential mediating role of the so-called metabolically healthy obesity status in the associations between traffic noise and cardiovascular outcomes represents an important knowledge gap.

### Mechanisms

The exact mechanisms between noise exposure and cardiovascular health are not completely understood. The most frequently mentioned mechanism is that chronic exposure to noise leads to activation of the autonomic and endocrine systems, and a subsequent cascade of stress hormones (i.e. catecholamines), which will be causing adverse changes in blood pressure, blood lipids and blood glucose.^2^ As discussed earlier, a novel pathway via adverse changes in systemic inflammation or vascular inflammation only came to light recently^38^ ^,^ ^39^ (Graphical Abstract). A study recently reported that the amygdala may be involved in processing the stress response via heightened arterial inflammation.^40^ There may also exist a potential social mechanism in light of some recent studies linking road traffic noise^41^ or noise annoyance^42^ with reduced levels of physical activity, the consequences of which may cause unfavourable changes in CVD biochemistry profiles.

### Strengths and limitations

The main strength of this study is the large study sample of >370 000 individuals in the main adjusted models, with detailed information on a variety of demographic, lifestyle, individual, and area-level socioeconomic variables. The study has limitations. The cross-sectional study design offers little support on causality and longitudinal studies are needed to strengthen the findings. Residual confounding from diet, physical activity, dyslipidaemia, family history, and other unmeasured factors may potentially bias our results. Light exposure at night, for which we did not have data, may potentially be another important confounding factor as it has been associated with the progression of carotid atherosclerosis^43^ and CVD hospitalization and death.^44^

Furthermore, as with all other studies of this type, exposure assignment of road traffic noise bears some uncertainty as time spent outside of home, layout of rooms in the house, window opening habits, noise sensitivity and indoor noise levels were not taken into account. Such exposure misclassification may have diluted our observed associations. *L*_night_ exposure likely has reduced misclassification as most people stay at home during night-time hours. However, our modelled estimates from using categorical *L*_night_ were similar as using *L*_den_. Our noise model at residential address did not specify a particular façade point, and therefore, it is likely that our estimated effects may have been underestimated. For instance, Foraster *et al.* ^7^ estimated that change in SBP was −0.20 mmHg (95% CI −1.25, 0.84) per 5 dB[A] of outdoor road traffic *L*_night_. The estimate increased to 0.36 mmHg (95% CI −0.06, 0.77) when outdoor road traffic *L*_night_ at bedroom façade was analysed and 0.72 mmHg (95% CI 0.29, 1.15) when indoor road traffic *L*_night_ in the bedroom was analysed. These findings highlight the importance of applying façade modelling in estimating noise exposures, which likely improves health effects estimation to a greater accuracy. Another limitation of our noise model is that it tended to over-estimate noise exposure at low levels due to the assumed national traffic flow baseline value but to under-estimate exposure for those heavily trafficked minor roads.^2^ Because of this, continuous noise estimates may be subject to more uncertainty and therefore we opted for categorical noise analyses, which may have relatively reduced misclassification.

The respective 0.77%, 0.49% and 0.79% increase in SBP, DBP and triglycerides observed in the main model equates to an approximate increase of 1.06 mmHg, 0.40 mmHg and 0.014 mmol/L for every 10 dB higher of road traffic noise. These effect sizes are small and potentially within the precision of instrumentation and random variation for an individual. Measurements were only taken at baseline visit, which did not truly reflect longer-term levels of these markers as measurement errors and intra-individual biological variability over time cannot be reliably accounted for without repeated measurements. A recent report from UK Biobank showed consistent mean values and a high self-correlation between baseline and repeated measurements among 20 000 participants for blood lipids and glycated haemoglobin.^45^ For an individual, clinical impact of such magnitude would be very small, if not negligible, despite statistical significance. At the population level, this may be particularly alarming given that currently across Europe 32 million people (nearly five million in UK) are exposed to road traffic noise >65 dB.^46^

In conclusion, this study provides evidence of long-term exposure to road traffic noise over 65 dB and elevated levels of CVD risk factors, particularly SBP and DBP. Future research with the consideration of both traffic noise and air pollution exposures is needed to provide further clarification on the multiple mechanisms between road traffic noise and CVD manifestation.

## Supplementary material

Supplementary material is available at *European Heart Journal* online.

### Data availability

The data underlying this article can be requested from UK Biobank (https://www.ukbiobank.ac.uk/).

## Supplementary Material

ehab121_Supplementary_Data
