# Supplementary material for: Road traffic noise and cardiovascular disease risk factors in UK Biobank
Source: Eur Heart J. 2021 Mar 18;42(21):2072–84. doi: 10.1093/eurheartj/ehab121 (PMC8169156; doi:10.1093/eurheartj/ehab121)
Supplement: ehab121_Supplementary_Data [file ehab121_supplementary_data.docx]

**Online Supplementary**

**Road Traffic Noise and Cardiovascular Disease Risk Factors in UK Biobank**

Zuzana Kupcikova, health consultant ^1,2^

Daniela Fecht, lecturer in geospatial health ^2^

Rema Ramakrishnan, biostatistician^3,4^

Charlotte Clark, epidemiologist/chartered psychologist^1^

Yutong Samuel Cai, senior epidemiologist ^2,3,4^ *

*1.Acoustics, Ove Arup & Partners, 13 Fitzroy Street, London, W1T 4BQ, UK*

*2.MRC Centre for Environment and Health, Department of Epidemiology and Biostatistics, School of Public Health, Imperial College London, London, UK*

*3.Nuffield Department of Women’s & Reproductive Health, University of Oxford, Oxford, UK*

*4.Deep Medicine Programme, Oxford Martin School, University of Oxford, Oxford, UK*

*** Corresponding Author: Dr Yutong Samuel Cai, Nuffield Department of Women’s & Reproductive Health, University of Oxford, Women's Centre (Level 3), John Radcliffe Hospital, OX3 9DU, UK. Email: yutong.cai@wrh.ox.ac.uk

**Appendix A**

**Table A1: The association between exposure to night -time (L_night_) road traffic noise and cardiovascular disease risk factors**

| Noise Exposure, L_night_ | N | Model 1  % change (95% CI) | N | Model 2  % change (95% CI) | N | Model 3  % change (95% CI) |
| --- | --- | --- | --- | --- | --- | --- |
| **Systolic Blood Pressure, mmHg** | 451, 132 |  | 378, 302 |  | 378, 302 |  |
| Low (≤45 dB[A]) |  | reference |  | reference |  | reference |
| Low-medium (>45 to ≤50 dB[A]) |  | **-0.31 (-0.39, -0.22)** |  | **-0.17 (-0.25, -0.08)** |  | -0.08 (-0.16, 0.00) |
| Medium-high (>50 to ≤55 dB[A]) |  | **-0.57 (-0.73, -0.41)** |  | **-0.19 (-0.35, -0.03)** |  | -0.11 (-0.27, 0.05) |
| High (>55 dB[A]) |  | **-0.30 (-0.46, -0.14)** |  | 0.05 (-0.11, 0.21) |  | **0.69 (0.52, 0.86)** |
| **Diastolic Blood Pressure, mmHg** | 450, 843 |  | 378, 073 |  | 378, 073 |  |
| Low (≤45 dB[A]) |  | reference |  | reference |  | reference |
| Low-medium (>45 to ≤50 dB[A]) |  | **-0.09 (-0.17, -0.01)** |  | **-0.13 (-0.21, -0.05)** |  | -0.08 (-0.16, 0.00) |
| Medium-high (>50 to ≤55 dB[A]) |  | **-0.27 (-0.42, -0.12)** |  | **-0.21 (-0.36, -0.05)** |  | -0.16 (-0.31, 0.00) |
| High (>55 dB[A]) |  | 0.07 (-0.08, 0.22) |  | 0.03 (-0.12, 0.19) |  | **0.42 (0.25, 0.58)** |
| **Triglycerides, mmol/L** | 462,346 |  | 389,392 |  | 389, 392 |  |
| Low (≤45 dB[A]) |  | reference |  | reference |  | reference |
| Low-medium (>45 to ≤50 dB[A]) |  | -0.33 (-0.65, -0.01) |  | -0.34 (-0.66, -0.02) |  | -0.09 (-0.42, 0.23) |
| Medium-high (>50 to ≤55 dB[A]) |  | **-0.85 (-1.47, -0.23)** |  | -0.79 (-1.40, -0.17) |  | -0.57 (-1.18, 0.05) |
| High (>55 dB[A]) |  | -0.40 (-1.01, 0.21) |  | -1.03 (-1.66, -0.41) |  | **0.75 (0.08, 1.42)** |
| **Glycated Haemoglobin, mmol/mol** | 449, 135 |  | 378, 890 |  | 378, 890 |  |
| Low (≤45 dB[A]) |  | reference |  | reference |  | reference |
| Low-medium (>45 to ≤50 dB[A]) |  | 0.07 (0.00, 0.15) |  | 0.06 (-0.01, 0.14) |  | 0.06 (-0.01, 0.14) |
| Medium-high (>50 to ≤55 dB[A]) |  | -0.05 (-0.20, 0.10) |  | -0.02 (-0.17, 0.13) |  | -0.02 (-0.17, 0.13) |
| High (>55 dB[A]) |  | **0.32 (0.18, 0.47)** |  | 0.09 (-0.06, 0.24) |  | 0.07 (-0.09, 0.23) |
| **C-Reactive Protein, mg/L** | 442, 544 |  | 373, 261 |  | 373, 261 |  |
| Low (≤45 dB[A]) |  | reference |  | reference |  | reference |
| Low-medium (>45 to ≤50 dB[A]) |  | 0.28 (-0.32, 0.88) |  | -0.52 (-1.09, 0.05) |  | -0.42 (-1.00, 0.16) |
| Medium-high (>50 to ≤55 dB[A]) |  | -0.45 (-1.60, 0.71) |  | **-1.15 (-2.24, -0.05)** |  | -1.06 (-2.17, 0.04) |
| High (>55 dB[A]) |  | **1.58 (0.41, 2.76)** |  | **-1.16 (-2.25, -0.06)** |  | -0.43 (-1.60, 0.75) |
| Noise Exposure, L_night_ | N | Model 1  Odds Ratio (95% CI) | N | Model 2  Odds Ratio (95% CI) | N | Model 3  Odds Ratio (95% CI) |
| **Self – Reported Hypertension** | 492,993 |  | 412,051 |  | 412,051 |  |
| Low (≤45 dB[A]) |  | reference |  | reference |  | reference |
| Low-medium (>45 to ≤50 dB[A]) |  | 1.00 (0.98, 1.01) |  | 0.99 (0.97, 1.00) |  | 0.98 (0.97, 1.00) |
| Medium-high (>50 to ≤55 dB[A]) |  | 0.99 (0.96, 1.01) |  | 0.97 (0.94, 1.00) |  | 0.97 (0.94, 1.00) |
| High (>55 dB[A]) |  | 1.03 (1.00, 1.06) |  | 0.99 (0.96, 1.02) |  | 0.95 (0.92, 0.98) |
| **Self – Reported Hypertension** | 492, 993 |  | 412, 051 |  | 412, 051 |  |
| Continuous L_night_ |  | 1.00 (1.00, 1.00) |  | 1.00 (1.00, 1.00) |  | 1.00 (0.99, 1.00) |

**Model 1:** unadjusted crude model. **Model 2:** fully adjusted model. Adjusted for sex, age, BMI, smoking status, alcohol intake frequency, Townsend deprivation index, household income, economic status, season of blood draw, length of time at residence**. Model 3:** Fully adjusted model (Model 2) + adjusted for NO_2_.  **Bold** represents significance at p<0.05

**Appendix B**

**Table B1: The association between road traffic noise and cardiovascular risk factors, unadjusted for BMI**

| Noise Exposure, L_den_ | N | Model 3 – BMI  % change (95% CI) |
| --- | --- | --- |
| **Systolic Blood Pressure, mmHg** | 379,725 |  |
| Low (≤55 dB[A]) |  | reference |
| Low-medium (>55 to ≤60 dB[A]) |  | -0.03 (-0.11, 0.06) |
| Medium-high (>60 to ≤65 dB[A]) |  | -0.04 (-0.21, 0.13) |
| High (>65 dB[A]) |  | 0.93 (0.75, 1.11) |
| **Diastolic Blood Pressure, mmHg** | 379,491 |  |
| Low (≤55 dB[A]) |  | reference |
| Low-medium (>55 to ≤60 dB[A]) |  | -0.00 (-0.09, 0.08) |
| Medium-high (>60 to ≤65 dB[A]) |  | -0.13 (-0.30, 0.04) |
| High (>65 dB[A]) |  | 0.71 (0.54, 0.88) |
| **Triglyceride, mmol/L** | 390,700 |  |
| Low (≤55 dB[A]) |  | reference |
| Low-medium (>55 to ≤60 dB[A]) |  | -0.06 (-0.40, 0.28) |
| Medium-high (>60 to ≤65 dB[A]) |  | -0.60 (-1.27, -0.09) |
| High (>65 dB[A]) |  | 1.83 (1.12, 2.55) |
| **Glycated Haemoglobin, mmol/mol** | 380,108 |  |
| Low (≤55 dB[A]) |  | reference |
| Low-medium (>55 to ≤60 dB[A]) |  | 0.06 (-0.02, 0.14) |
| Medium-high (>60 to ≤65 dB[A]) |  | -0.05 (-0.21, 0.10) |
| High (>65 dB[A]) |  | 0.26 (0.10, 0.43) |
| **C-Reactive Protein, mg/L** | 374,426 |  |
| Low (≤55 dB[A]) |  | reference |
| Low-medium (>55 to ≤60 dB[A]) |  | -0.29 (-0.93, 0.34) |
| Medium-high (>60 to ≤65 dB[A]) |  | -1.27 (-2.33, -0.19) |
| High (>65 dB[A]) |  | 2.35 (1.21, 3.50) |
| Noise Exposure, L_den_ | N | Model 3 – BMI  Odds Ratio (95% CI) |
| **Self-Reported Hypertension** | 415, 750 |  |
| Low (≤55 dB[A]) |  | reference |
| Low-medium (>55 to ≤60 dB[A]) |  | 0.99 (0.80, 1.01) |
| Medium-high (>60 to ≤65 dB[A]) |  | 0.98 (0.57, 1.01) |
| High (>65 dB[A]) |  | 0.98 (0.95, 1.02) |
| **Self – Reported Hypertension** | 415, 750 |  |
| Continuous L_den_ |  | 0.99 (1.00, 1.00) |

**Model 3 - BMI** - Fully adjusted model (Model 2) + adjusted for NO_2_, except for adjustment for BMI. **Bold** represents significance at p<0.05.

**Appendix C**

**Table C1: The association between exposure to road traffic noise and cardiovascular disease risk factors, adjusted for ever had diabetes or hypertension**

| Noise Exposure, L_den_ | N | Model 3  % change (95% CI) | N | Model 3 + EHDH  % change (95% CI) |
| --- | --- | --- | --- | --- |
| **Systolic Blood Pressure, mmHg** | 378,302 |  | 376, 948 |  |
| Low (≤55 dB[A]) |  | reference |  | reference |
| Low-medium (>55 to ≤60 dB[A]) |  | -0.04 (-0.12, 0.05) |  | -0.02 (-0.10, 0.06) |
| Medium-high (>60 to ≤65 dB[A]) |  | -0.03 (-0.20, 0.13) |  | -0.02 (-0.18, 0.15) |
| High (>65 dB[A]) |  | **0.77 (0.60, 0.95)** |  | **0.83 (0.66, 1.00)** |
| **Diastolic Blood Pressure, mmHg** | 378,073 |  | 376, 720 |  |
| Low (≤55 dB[A]) |  | reference |  | reference |
| Low-medium (>55 to ≤60 dB[A]) |  | -0.02 (-0.10, 0.06) |  | 0.00 (-0.08, 0.08) |
| Medium-high (>60 to ≤65 dB[A]) |  | -0.11 (-0.27, 0.05) |  | -0.10 (-0.26, 0.06) |
| High (>65 dB[A]) |  | **0.49 (0.32, 0.65)** |  | **0.54 (0.37, 0.70)** |
| **Triglyceride, mmol/L** | 389,392 |  | 388, 029 |  |
| Low (≤55 dB[A]) |  | reference |  | reference |
| Low-medium (>55 to ≤60 dB[A]) |  | -0.10 (-0.43, 0.22) |  | -0.08 (-0.40, 0.25) |
| Medium-high (>60 to ≤65 dB[A]) |  | -0.57 (-1.22, 0.08) |  | -0.55 (-1.20, 0.11) |
| High (>65 dB[A]) |  | **0.79 (0.11, 1.47)** |  | **0.83 (0.15, 1.51)** |
| **Glycated Haemoglobin, mmol/mol** | 378,890 |  | 377, 593 |  |
| Low (≤55 dB[A]) |  | reference |  | reference |
| Low-medium (>55 to ≤60 dB[A]) |  | 0.05 (-0.03, 0.13) |  | 0.04 (-0.03, 0.11) |
| Medium-high (>60 to ≤65 dB[A]) |  | -0.05 (-0.20, 0.11) |  | -0.05 (-0.20, 0.10) |
| High (>65 dB[A]) |  | 0.12 (-0.04, 0.28) |  | 0.11 (-0.04, 0.27) |
| **C-Reactive Protein, mg/L** | 373,261 |  | 371, 986 |  |
| Low (≤55 dB[A]) |  | reference |  | reference |
| Low-medium (>55 to ≤60 dB[A]) |  | -0.36 (-0.94, 0.22) |  | -0.36 (-0.94, 0.22) |
| Medium-high (>60 to ≤65 dB[A]) |  | -1.03 (-2.18, 0.13) |  | -1.07 (-2.22, 0.10) |
| High (>65 dB[A]) |  | -0.27 (-1.46, 0.93) |  | -0.28 (-1.47, 0.93) |
| Noise Exposure, L_den_ | N | Model 3  Odds Ratio (95% CI) | N | Model 3 + EHD  Odds Ratio (95% CI) |
| **Self – Reported Hypertension** | 413,845 |  | 412, 982 |  |
| Low (≤55 dB[A]) |  | reference |  | reference |
| Low-medium (>55 to ≤60 dB[A]) |  | 0.99 (0.98, 1.01) |  | 0.99 (0.98, 1.01) |
| Medium-high (>60 to ≤65 dB[A]) |  | 0.98 (0.95, 1.01) |  | 0.98 (0.95, 1.01) |
| High (>65 dB[A]) |  | **0. 95 (0.92, 0.98)** |  | 0.95 (0.92, 0.99) |
| **Self – Reported Hypertension** | 413,845 |  | 412, 982 |  |
| Continuous L_den_ |  | 1.00 (0.99, 1.00) |  | 1.00 (0.99, 1.00) |

**Model 3 -** Fully adjusted model (Model 2) + adjusted for NO_2._ **Model 3 + ever had diabetes or hypertension (EHDH)** – Fully adjusted model (Model 2) + adjusted for NO_2_, plus adjustment for ever had diabetes and hypertension. **Model 3 + ever had diabetes (EHD)** – Fully adjusted model (Model 2) + adjusted for NO_2_, plus adjustment for ever had diabetes. **Bold** represents significance at p<0.05.

| Noise Exposure, L_den_ | N | Model 1  % change (95% CI) | N | Model 2  % change (95% CI) | N | Model 3  % change (95% CI) | N | Model 4  % change (95% CI) |
| --- | --- | --- | --- | --- | --- | --- | --- | --- |
| **Systolic Blood Pressure, mmHg** | 502,521 |  | 502,521 |  | 502,521 |  | 502,521 |  |
| Low (≤55 dB[A]) |  | reference |  | reference |  | reference |  | reference |
| Low-medium (>55 to ≤60 dB[A]) |  | **-0.30 (-0.39, -0.22)** |  | -0.07 (-0.15, 0.01) |  | 0.00 (-0.07, 0.08) |  | -0.02 (-0.09, 0.06) |
| Medium-high (>60 to ≤65 dB[A]) |  | **-0.52 (-0.68, -0.35)** |  | -0.15 (-0.30, 0.01) |  | -0.06 (-0.22, 0.09) |  | -0.06 (-0.21, 0.10) |
| High (>65 dB[A]) |  | **-0.23 (-0.39, -0.07)** |  | 0.05 (-0.10, 0.20) |  | **0.73 (0.57, 0.89)** |  | **0.28 (0.13, 0.44)** |
| **Diastolic Blood Pressure, mmHg** | 456,989 |  | 456,989 |  | 456,989 |  | 456,989 |  |
| Low (≤55 dB[A]) |  | reference |  | reference |  | reference |  | reference |
| Low-medium (>55 to ≤60 dB[A]) |  | -0.04 (-0.11, 0.04) |  | -0.04 (-0.11, 0.04) |  | 0.01 (-0.07, 0.08) |  | 0.01 (-0.07, 0.08) |
| Medium-high (>60 to ≤65 dB[A]) |  | **-0.24 (-0.40, -0.08)** |  | **-0.19 (-0.34, -0.04)** |  | -0.14 (-0.28, 0.01) |  | -0.11 (-0.26, 0.04) |
| High (>65 dB[A]) |  | 0.12 (-0.03, 0.27) |  | 0.03 (-0.11, 0.18) |  | **0.45 (0.30, 0.61)** |  | **0.23 (0.08, 0.38)** |
| **Triglycerides, mmol/L** | 502,521 |  | 502,521 |  | 502,521 |  | 502,521 |  |
| Low (≤55 dB[A]) |  | reference |  | reference |  | reference |  | reference |
| Low-medium (>55 to ≤60 dB[A]) |  | -0.29 (-0.61, 0.04) |  | -0.27 (-0.56, 0.03) |  | -0.05 (-0.35, 0.24) |  | -0.16 (-0.46, 0.14) |
| Medium-high (>60 to ≤65 dB[A]) |  | **-0.89 (-1.54, -0.23)** |  | **-0.76 (-1.37, -0.16)** |  | -0.52 (-1.12, 0.09) |  | -0.60 (-1.20, 0.01) |
| High (>65 dB[A]) |  | -0.32 (-0.94, 0.31) |  | **-1.08 (-1.67, -0.50)** |  | **0.86 (0.23, 1.48)** |  | **-0.66 (-1.25, -0.06)** |
| **Glycated Haemoglobin, mmol/mol** | 502,521 |  | 502,521 |  | 378,890 |  | 502,521 |  |
| Low (≤55 dB[A]) |  | reference |  | reference |  | reference |  | reference |
| Low-medium (>55 to ≤60 dB[A]) |  | 0.06 (-0.02, 0.14) |  | 0.05 (-0.03, 0.12) |  | 0.04 (-0.04, 0.11) |  | 0.05 (-0.02, 0.13) |
| Medium-high (>60 to ≤65 dB[A]) |  | -0.09 (-0.24, 0.07) |  | -0.08 (-0.22, 0.07) |  | -0.09 (-0.23, 0.06) |  | -0.07 (-0.21, 0.08) |
| High (>65 dB[A]) |  | **0.39 (0.24, 0.55)** |  | **0.18 (0.04, 0.32)** |  | 0.11 (-0.04, 0.25) |  | **0.21 (0.07, 0.36)** |
| **C-Reactive Protein, mg/L** | 502,521 |  | 502,521 |  | 502,521 |  | 502,521 |  |
| Low (≤55 dB[A]) |  | reference |  | reference |  | reference |  | reference |
| Low-medium (>55 to ≤60 dB[A]) |  | 0.41 (-0.19, 1.02) |  | -0.39 (-0.92, 0.14) |  | -0.30 (-0.84, 0.24) |  | -0.40 (-0.93, 0.14) |
| Medium-high (>60 to ≤65 dB[A]) |  | -0.46 (-1.65, 0.73) |  | **-1.13 (-2.18, -0.08)** |  | -1.02 (-2.08, 0.03) |  | **-1.14 (-2.19, -0.08)** |
| High (>65 dB[A]) |  | **1.84 (0.68, 3.01)** |  | **-1.02 (-2.03, -0.02)** |  | -0.19 (-1.27, 0.91) |  | -1.04 (-2.08, 0.00) |
| Noise Exposure, L_den_ | N | Model 1  Odds Ratio (95% CI) | N | Model 2  Odds Ratio (95% CI) | N | Model 3  Odds Ratio (95% CI) | N | Model 4  Odds Ratio (95% CI) |
| **Self-Reported Hypertension** | 502,521 |  | 502,521 |  | 502,521 |  | 502,521 |  |
| Low (≤55 dB[A]) |  | reference |  | reference |  | reference |  | reference |
| Low-medium (>55 to ≤60 dB[A]) |  | 1.00 (0.99, 1.01) |  | 1.00 (0.99, 1.01) |  | 1.00 (0.98, 1.01) |  | 1.00 (0.98, 1.01) |
| Medium-high (>60 to ≤65 dB[A]) |  | 1.00 (0.97, 1.03**)** |  | 1.00 (0.97, 1.03) |  | 0.99 (0.96, 1.02) |  | 0.99 (0.96, 1.02) |
| High (>65 dB[A]) |  | 1.03 (1.00, 1.06) |  | 1.03 (1.00, 1.06) |  | **0. 95 (0.92, 0.98)** |  | 0.98 (0.95, 1.00) |

**Appendix D: Multiple Imputation Analysis**

**Table D1: The association between exposure to road traffic noise and cardiovascular disease risk factors using an imputed dataset**

**Model 1:** unadjusted crude model. **Model 2:** fully adjusted model. Adjusted for sex, age, BMI, smoking status, alcohol intake frequency, Townsend deprivation index, household income, economic status, season of blood draw, length of time at residence**.** **Model 3:** Fully adjusted model (Model 2) + adjusted for NO_2_. Model 4: Fully adjusted model **(Model2)** + adjusted for PM_2.5_ **Bold** represents significance at p<0.05.

**Appendix E**

**Table E1: The association between exposure road traffic noise and cardiovascular disease risk factors, with reference group set to** ≤**52dB[A]**

| Noise Exposure, L_den_ | N | Model 1  % change (95% CI) | N | Model 2  % change (95% CI) | N | Model 3  % change (95% CI) |
| --- | --- | --- | --- | --- | --- | --- |
| **Systolic Blood Pressure, mmHg** | 451, 132 |  | 379, 973 |  | 379, 973 |  |
| Very – low (≤52 dB[A]) |  | reference |  | reference |  | reference |
| Low (>52 to ≤55 dB[A]) |  | **-0.21 (-0.35, -0.07)** |  | **-0.25 (-0.39, -0.11)** |  | **-0.15 (-0.29, -0.01)** |
| Low - medium (>55 to ≤58dB[A]) |  | **-0.49 (-0.63, -0.34)** |  | **-0.32 (-0.47, -0.18)** |  | **-0.16 (-0.31, -0.02)** |
| Medium (>58 to ≤61 dB[A]) |  | **-0.56 (-0.74, -0.37)** |  | **-0.28 (-0.46, -0.10)** |  | -0.16 (-0.35, 0.02) |
| Medium - high (>61 to ≤ 64 dB[A]) |  | **-0.72 (-0.97, -0.47)** |  | **-0.34 (-0.58, -0.09)** |  | -0.17 (-0.42, 0.07) |
| High (>64 dB[A]) |  | **-0.42 (-0.61, -0.22)** |  | -0.09 (-0.28, 0.10) |  | **0.60 (0.40, 0.81)** |
| **Diastolic Blood Pressure, mmHg** | 451, 144 |  | 379,984 |  | 379,984 |  |
| Very – low (≤52 dB[A]) |  | reference |  | reference |  | reference |
| Low (>52 to ≤55 dB[A]) |  | -0.13 (-0.26, 0.00) |  | **-0.14 (-0.28, -0.01)** |  | -0.08 (-0.22, 0.05) |
| Low - medium (>55 to ≤58dB[A]) |  | **-0.15 (-0.28, -0.01)** |  | **-0.19 (-0.33, -0.05)** |  | -0.09 (-0.23, 0.05) |
| Medium (>58 to ≤61 dB[A]) |  | **-0.21 (-0.38, -0.04)** |  | -0.17 (-0.35, 0.01) |  | -0.10 (-0.28, 0.08) |
| Medium - high (>61 to ≤ 64 dB[A]) |  | **-0.33 (-0.57, -0.10)** |  | **-0.29 (-0.53, -0.05)** |  | -0.19 (-0.43, 0.05) |
| High (>64 dB[A]) |  | 0.00 (-0.18, 0.18) |  | -0.03 (-0.21, 0.16) |  | **0.40 (0.21, 0.60)** |
| **Triglycerides, mmol/L** | 462, 346 |  | 391, 094 |  | 391,094 |  |
| Very – low (≤52 dB[A]) |  | reference |  | reference |  | reference |
| Low (>52 to ≤55 dB[A]) |  | 0.07 (-0.47, 0.62) |  | -0.35 (-0.88, 0.19) |  | -0.06 (-0.60, 0.48) |
| Low - medium (>55 to ≤58dB[A]) |  | -0.21 (-0.77, 0.35) |  | **-0.57 (-1.12, -0.01)** |  | -0.11 (-0.67, 0.46) |
| Medium (>58 to ≤61 dB[A]) |  | -0.44 (-1.14, 0.27) |  | **-0.72 (-1.42, -0.01)** |  | -0.38 (-1.09, 0.33) |
| Medium - high (>61 to ≤ 64 dB[A]) |  | -0.68 (-1.64, 0.28) |  | **-1.29 (-2.23, -0.34)** |  | -0.84 (-1.78, 0.12) |
| High (>64 dB[A]) |  | -0.41 (-1.14, 0.34) |  | **-1.31 (-2.05, -0.57)** |  | 0.64 (-0.14, 1.44) |
| **Glycated Haemoglobin, mmol/mol** | 449,135 |  | 380,510 |  | 380,510 |  |
| Very – low (≤52 dB[A]) |  | reference |  | reference |  | reference |
| Low (>52 to ≤55 dB[A]) |  | **0.22 (0.09, 0.35)** |  | 0.10 (-0.02, 0.23) |  | 0.10 (-0.03,0.22) |
| Low - medium (>55 to ≤58dB[A]) |  | **0.23 (0.10, 0.36)** |  | **0.16 (0.03, 0.29)** |  | **0.15 (0.02, 0.28)** |
| Medium (>58 to ≤61 dB[A]) |  | 0.12 (-0.05, 0.29) |  | 0.05 (-0.12, 0.22) |  | 0.04 (-0.13, 0.21) |
| Medium - high (>61 to ≤ 64 dB[A]) |  | 0.13 (-0.10, 0.36) |  | 0.06 (-0.17, 0.29) |  | 0.05 (-0.18, 0.28) |
| High (>64 dB[A]) |  | **0.48 (0.30, 0.65)** |  | 0.17 (-0.01, 0.35) |  | 0.12 (-0.06, 0.31) |
| **C-Reactive Protein, mg/L** | 442,544 |  | 378, 877 |  | 374, 877 |  |
| Very – low (≤52 dB[A]) |  | reference |  | reference |  | reference |
| Low (>52 to ≤55 dB[A]) |  | 0.02 (-0.98, 1.04) |  | -0.89 (-1.84, 0.07) |  | -0.79 (-1.74, 0.17) |
| Low - medium (>55 to ≤58dB[A]) |  | 0.18 (-0.87, 1.24) |  | **-1.29 (-2.28, -0.30)** |  | **-1.13 (-2.13, -0.13)** |
| Medium (>58 to ≤61 dB[A]) |  | **0.57 (-0.75, -1.88)** |  | -0.99 (-2.23, 0.28) |  | -0.87 (-2.13, 0.40) |
| Medium - high (>61 to ≤ 64 dB[A]) |  | -0.44 (-2.22, 1.36) |  | -1.70 (-3.37, 0.00) |  | -1.54 (-3.22, 0.17) |
| High (>64 dB[A]) |  | 1.37 (-0.03, 2.76) |  | **-1.91 (-3.21, -0.59)** |  | -1.22 (-2.59, 0.17) |
| Noise Exposure, L_den_ | N | Model 1  Odds Ratio (95% CI) | N | Model 2  Odds Ratio (95% CI) | N | Model 3  Odds Ratio (95% CI) |
| **Self – reported Hypertension** | 492, 993 |  | 413, 845 |  | 413, 845 |  |
| Very – low (≤52 dB[A]) |  | reference |  | reference |  | reference |
| Low (>52 to ≤55 dB[A]) |  | 1.01 (0.99, 1.03) |  | 1.00 (0.97, 1.03) |  | 0.99 (0.97, 1.02) |
| Low - medium (>55 to ≤58dB[A]) |  | 1.01 (0.99, 1.04) |  | 1.00 (0.97, 1.03) |  | 0.99 (0.96, 1.02) |
| Medium (>58 to ≤61 dB[A]) |  | 1.00 (0.97, 1.03) |  | 0.98 (0.95, 1.02) |  | 0.98 (0.94, 1.01) |
| Medium - high (>61 to ≤ 64 dB[A]) |  | 1.01 (0.97, 1.05) |  | 0.98 (0.93, 1.03) |  | 0.97 (0.92, 1.02) |
| High (>64 dB[A]) |  | 1.04 (1.00, 1.07) |  | 0.99 (0.96, 1.03) |  | **0.94 (0.91, 0.98)** |

**Model 1:** unadjusted crude model. **Model 2:** fully adjusted model. Adjusted for sex, age, BMI, smoking status, alcohol intake frequency, Townsend deprivation index, household income, economic status, season of blood draw, length of time at residence**. Model 3:** Fully adjusted model (Model 2) + adjusted for NO_2_.  **Bold** represents significance at p<0.05

**Appendix F: Effect Modification Results**

**Table F1: P-interaction values for the association between the exposure to road traffic noise and cardiovascular risk factors**

| Model 3, Noise Exposure, L_den_ | Sex    P_interaction_ | Age  P_interaction_ | Time at Residence  P_interaction_ | Townsend  Deprivation Index  P_interaction_ |
| --- | --- | --- | --- | --- |
| **Systolic Blood Pressure, mmHg** |  |  |  |  |
| Low (≤55 dB[A]) | reference | reference | reference | reference |
| Low-medium (>55 to ≤60 dB[A]) | **0.00 (+)** | 0.09 (-) | 0.78 (-) | 0.69 (+) |
| Medium-high (>60 to ≤65 dB[A]) | **0.00 (+)** | 0.34 (+) | 0.63 (-) | 0.59 (+) |
| High (>65 dB[A]) | 0.81 (+) | 0.27 (-) | 0.21 (-) | 0.84 (+) |
| **Diastolic Blood Pressure, mmHg** |  |  |  |  |
| Low (≤55 dB[A]) | reference | reference | reference | reference |
| Low-medium (>55 to ≤60 dB[A]) | 0.44 (+) | 0.67 (+) | 0.95 (-) | 0.96 (+) |
| Medium-high (>60 to ≤65 dB[A]) | 0.19 (+) | 0.14 (+) | 0.80 (-) | 0.89(+) |
| High (>65 dB[A]) | 0.38 (-) | 1.00 (-) | 0.69 (-) | 0.33 (-) |
| **Triglyceride, mmol/L** |  |  |  |  |
| Low (≤55 dB[A]) | reference | reference | reference | reference |
| Low-medium (>55 to ≤60 dB[A]) | 0.06 (+) | 0.79 (+) | 0.80 (+) | 0.70 (+) |
| Medium-high (>60 to ≤65 dB[A]) | 0.08 (+) | 0.19 (-) | 0.60 (-) | 0.90 (+) |
| High (>65 dB[A]) | 0.62 (+) | 0.99 (+) | 0.44 (-) | 0.22 (+) |
| **Glycated Haemoglobin, mmol/mol** |  |  |  |  |
| Low (≤55 dB[A]) | reference | reference | reference | reference |
| Low-medium (>55 to ≤60 dB[A]) | **0.04** (+) | **0.03 (+)** | 0.24 (+) | 0.45 (-) |
| Medium-high (>60 to ≤65 dB[A]) | **0.00** (+) | 0.58 (+) | 0.67 (-) | 0.67 (-) |
| High (>65 dB[A]) | **0.02** (+) | 0.21 (+) | 0.21 (-) | 0.74 (+) |
| **C- Reactive Protein, mg/L** |  |  |  |  |
| Low (≤55 dB[A]) | reference | reference | reference | reference |
| Low-medium (>55 to ≤60 dB[A]) | **0.04** (+) | 0.05 (+) | 0.22 (+) | 0.42 (+) |
| Medium-high (>60 to ≤65 dB[A]) | 0.06 (+) | 0.80 (-) | 0.30 (-) | 0.70 (+) |
| High (>65 dB[A]) | 0.97 (+) | 0.93 (+) | 0.44 (-) | 0.11 (+) |
| **Self – Reported Hypertension** |  |  |  |  |
| Low (≤55 dB[A]) | reference | reference | reference | reference |
| Low-medium (>55 to ≤60 dB[A]) | 0.11 (+) | 0.52 (+) | 0.57 (+) | 0.62 (-) |
| Medium-high (>60 to ≤65 dB[A]) | 0.10 (+) | 0.64 (-) | **0.00 (-)** | 0.13 (+) |
| High (>65 dB[A]) | 0.66 (+) | 0.79 (-) | 0.63 (+) | 0.57 (+) |

Interaction terms including sex, age, time at residence and Townsend deprivation index were added to Model 3 in the form of exposure*modifier. **Model 3:** Fully adjusted model (Model 2) + adjusted for NO_2_. **Bold** represents significance at p<0.05

**Table F2: P-interaction values for the interaction between road traffic noise and income for the association between exposure to road traffic noise and cardiovascular risk factors.**

|  | Average Total Household Income Before Tax | | | | |
| --- | --- | --- | --- | --- | --- |
| Model 3, Noise Exposure, L_den_ | *<£18,000*  P_interaction_ | *£18,000 to £30,999*  P_interaction_ | *£31,000 to £51,999*  P_interaction_ | *£52,000 to £100,000*  P_interaction_ | *>£100,000*  P_interaction_ |
| **Systolic Blood Pressure, mmHg** |  |  |  |  |  |
| Low (≤55 dB[A]) | reference | reference | reference | reference | reference |
| Low-medium (>55 to ≤60 dB[A]) | reference | 0.07 (-) | 0.237 (-) | **0.04 (-)** | 0.21 (-) |
| Medium-high (>60 to ≤65 dB[A]) | reference | 0.40 (-) | 0.39 (-) | 0.27 (-) | **0.04 (-)** |
| High (>65 dB[A]) | reference | 0.23 (-) | 0.05 (-) | 0.21 (-) | 0.66 (+) |
| **Diastolic Blood Pressure, mmHg** |  |  |  |  |  |
| Low (≤55 dB[A]) | reference | Reference | Reference | Reference | reference |
| Low-medium (>55 to ≤60 dB[A]) | reference | 0.11 (-) | 0.64 (-) | **0.03 (-)** | **0.05 (-)** |
| Medium-high (>60 to ≤65 dB[A]) | reference | 0.58 (+) | 0.92 (-) | 0.96 (-) | **0.02 (-)** |
| High (>65 dB[A]) | reference | 0.51 (-) | 0.72 (-) | 0.90 (-) | 0.81 (-) |
| **Triglyceride, mmol/L** |  |  |  |  |  |
| Low (≤55 dB[A]) | reference | Reference | Reference | Reference | Reference |
| Low-medium (>55 to ≤60 dB[A]) | reference | 0.07 (+) | 0.24 (+) | **0.01 (+)** | 0.09 (+) |
| Medium-high (>60 to ≤65 dB[A]) | reference | 0.77 (+) | 0.38 (+) | 0.20 (+) | 0.07 (+) |
| High (>65 dB[A]) | reference | 0.67 (+) | 0.60 (+) | 0.62 (+) | 0.94 (-) |
| **Glycated Haemoglobin, mmol/mol** |  |  |  |  |  |
| Low (≤55 dB[A]) | reference | Reference | Reference | Reference | Reference |
| Low-medium (>55 to ≤60 dB[A]) | reference | 0.90 (-) | 0.18 (-) | 0.15 (-) | 0.24 (-) |
| Medium-high (>60 to ≤65 dB[A]) | reference | 0.11 (-) | 0.10 (-) | 0.67 (-) | **0.03 (-)** |
| High (>65 dB[A]) | reference | 0.17 (+) | 0.38 (-) | 0.82 (-) | 0.09 (-) |
| **C- Reactive Protein, mg/L** |  |  |  |  |  |
| Low (≤55 dB[A]) | reference | Reference | Reference | Reference | Reference |
| Low-medium (>55 to ≤60 dB[A]) | reference | 0.25 (-) | 0.16 (-) | 0.58 (-) | **0.00 (-)** |
| Medium-high (>60 to ≤65 dB[A]) | reference | 0.50 (+) | 0.93 (-) | 0.18 (+) | 0.44 (+) |
| High (>65 dB[A]) | reference | 0.77 (-) | 0.66 (-) | 0.45 (+) | 0.93 (-) |
| **Self – Reported Hypertension** |  |  |  |  |  |
| Low (≤55 dB[A]) | reference | Reference | Reference | Reference | Reference |
| Low-medium (>55 to ≤60 dB[A]) | reference | 0.15 (+) | 0.92 (-) | 0.78 (+) | 0.15 (+) |
| Medium-high (>60 to ≤65 dB[A]) | reference | 0.62 (-) | 0.18 (-) | 0.06 (-) | 0.60 (+) |
| High (>65 dB[A]) | reference | 0.57 (-) | 0.26 (-) | 0.20 (-) | 0.99 (+) |

An interaction term including income was added to Model 3 in the form of exposure*modifier. **Model 3:** Fully adjusted model (Model 2) + adjusted for NO_2_. **Bold** represents significance at p<0.05

**Table F3: The association between exposure to road traffic noise and cardiovascular risk factors, stratified by sex**

| Model 3, Noise Exposure, L_den_ | P_Interaction_ | N | Males  % change (95% CI) | N | Females  % change (95% CI) |
| --- | --- | --- | --- | --- | --- |
| **Systolic Blood Pressure, mmHg** |  | 180,066 |  | 198,638 |  |
| Low (≤55 dB[A]) | reference |  | reference |  | reference |
| Low-medium (>55 to ≤60 dB[A]) | **0.00 (+)** |  | 0.10 (-0.06, 0.22) |  | -0.13 (-0.25, 0.02) |
| Medium-high (>60 to ≤65 dB[A]) | **0.00 (+)** |  | 0.02 (0.00, 0.46) |  | **-0.28 (-0.52, -0.05)** |
| High (>65 dB[A]) | 0.81 (+) |  | **0.72 (0.48, 0.96)** |  | **0.77 (0.52, 1.02)** |
| **Glycated Haemoglobin, mmol/mol** |  | 179,234 |  | 200,037 |  |
| Low (≤55 dB[A]) | reference |  | reference |  | reference |
| Low-medium (>55 to ≤60 dB[A]) | **0.04** (+) |  | 0.06 (-0.06, 0.18) |  | 0.04 (-0.06, 0.14) |
| Medium-high (>60 to ≤65 dB[A]) | **0.00** (+) |  | 0.17(-0.07, 0.40) |  | **-0.25 (-0.45, -0.05)** |
| High (>65 dB[A]) | **0.02** (+) |  | 0.18 (-0.06, 0.42) |  | 0.03 (-0.18, 0.23) |
| **C- Reactive Protein, mg/L** |  | 178,674 |  | 194,973 |  |
| Low (≤55 dB[A]) | reference |  | reference |  | reference |
| Low-medium (>55 to ≤60 dB[A]) | **0.04** (+) |  | -0.10 (-0.94, 0.74) |  | -0.59 (-1.39, 0.22) |
| Medium-high (>60 to ≤65 dB[A]) | 0.06 (+) |  | -0.52 (-2.17, 1.16) |  | -1.41 (-3.00, 0.20) |
| High (>65 dB[A]) | 0.97 (+) |  | -1.09 ( -2.77, 0.63) |  | 0.51 (-1.16, 2.21) |

**Model 3:** Fully adjusted model (Model 2) + adjusted for NO_2_. **Bold** represents significance at p<0.05.

**Table F4: The association between exposure to road traffic noise and glycated haemoglobin, stratified by age**

| Model 3, Noise Exposure, L_den_ | P*_Interaction_* | N | Age ≥ 65  % change (95% CI) | N | Age < 65  % change (95% CI) |
| --- | --- | --- | --- | --- | --- |
| **Glycated Haemoglobin, mmol/mol** |  | 66,555 |  | 312,716 |  |
| Low (≤55 dB[A]) | reference |  | reference |  | reference |
| Low-medium (>55 to ≤60 dB[A]) | **0.03 (+)** |  | 0.13 (-0.06, 0.32) |  | -0.01 (-0.10, 0.07) |
| Medium-high (>60 to ≤65 dB[A]) | 0.58 (+) |  | -0.01 (-0.40, 0.37) |  | -0.07 (-0.24, 0.11) |
| High (>65 dB[A]) | 0.21 (+) |  | 0.27 (-0.13, 0.66) |  | **0.21 (0.03, 0.39)** |

**Model 3:** Fully adjusted model (Model 2) + adjusted for NO_2_. **Bold** represents significance at p<0.05.

**Table F5: The association between exposure to road traffic noise and self-reported hypertension, stratified by time at residence**

| Model 3, Noise Exposure, L_den_ | P*_Interaction_* | N | Time at residence >10  Odds Ratio (95% CI) | N | Time at residence ≤10  Odds Ratio (95% CI) |
| --- | --- | --- | --- | --- | --- |
| **Self – Reported Hypertension** |  | 261,886 |  | 151,959 |  |
| Low (≤55 dB[A]) | reference |  | reference |  | reference |
| Low-medium (>55 to ≤60 dB[A]) | 0.57 (+) |  | 0.99 (0.97, 1.01) |  | 1.00 (0.97, 1.02) |
| Medium-high (>60 to ≤65 dB[A]) | **0.00 (-)** |  | **0.95 (0.91, 0.98)** |  | 1.04 (0.98, 1.09) |
| High (>65 dB[A]) | 0.63 (+) |  | **0.95 (0.91, 0.99)** |  | 0.95 (0.89, 1.00) |
| **Self – Reported Hypertension** |  | 261,886 |  | 151,959 |  |
| Continuous L_den_ | 0.50 |  | 1.00 (0.99, 1.00) |  | 1.00 (0.99, 1.00) |

**Model 3:** Fully adjusted model (Model 2) + adjusted for NO_2_. **Bold** represents significance at p<0.05.

|  |  | Average Total Household Income Before Tax | | | | | | | | |
| --- | --- | --- | --- | --- | --- | --- | --- | --- | --- | --- |
| Model 3, Noise Exposure, L_den_ | N | *<£18,000*  % change (95% CI) | N | *£18,000 to £30,999*  % change (95% CI) | N | *£31,000 to £51,999*  % change (95% CI) | N | *£52,000 to £100,000*  % change (95% CI) | N | *>£100,000*  % change (95% CI) |
| **Systolic Blood Pressure, mmHg** | 84,747 |  | 96,352 |  | 99,011 |  | 77,555 |  | 20,637 |  |
| Low (≤55 dB[A]) |  | reference |  | reference |  | reference |  | reference |  | reference |
| Low-medium (>55 to ≤60 dB[A]) |  | 0.13 ( -0.05, 0.32) |  | -0.12 (-0.30, 0.05) |  | -0.03 (-0.19, 0.14) |  | -0.10 (-0.28, 0.08) |  | -0.04 (-0.38, 0.30) |
| Medium-high (>60 to ≤65 dB[A]) |  | 0.16 (-0.20, 0.52) |  | -0.06 (-0.40, 0.29) |  | -0.04 (-0.36, 0.29) |  | -0.07 (-0.43, 0.29) |  | -0.53 (-1.16, 0.10) |
| High (>65 dB[A]) |  | **0.94 (0.56, 1.33)** |  | **0.72 (0.37, 1.08)** |  | **0.52 (0.18, 0.86)** |  | **0.72 (0.35, 1.09)** |  | **1.29 (0.61, 1.97)** |
| **Diastolic Blood Pressure, mmHg** | 84,689 |  | 96,290 |  | 98,954 |  | 77,516 |  | 20,624 |  |
| Low (≤55 dB[A]) |  | reference |  | reference |  | reference |  | reference |  | reference |
| Low-medium (>55 to ≤60 dB[A]) |  | 0.09 (-0.09, 0.26) |  | -0.09 (-0.25, 0.07) |  | 0.08 (-0.08, 0.23) |  | **-0.10 (-0.27, 0.08)** |  | -0.05 (-0.40, 0.29) |
| Medium-high (>60 to ≤65 dB[A]) |  | -0.14 (-0.49, 0.20) |  | 0.01 (-0.31, 0.33) |  | -0.08 (-0.39, 0.23) |  | -0.04 (-0.39, 0.31) |  | **-0.76 (-1.38, -0.13)** |
| High (>65 dB[A]) |  | **0.38 (0.01, 0.74)** |  | **0.34 (0.01, 0.68)** |  | **0.46 (0.13, 0.79)** |  | **0.60 (0.25, 0.97)** |  | **0.72 (0.05, 1.39)** |
| **Triglyceride, mmol/L** |  |  |  |  |  |  |  |  |  |  |
| Low (≤55 dB[A]) | 87,221 | reference | 99,182 | reference | 102,110 | reference | 79,798 | reference | 21,081 | reference |
| Low-medium (>55 to ≤60 dB[A]) |  | -0.54 (-1.22, 0.16) |  | 0.09 (-0.55, 0.74) |  | -0.37 (-0.99, 0.27) |  | **0.58 (-0.14, 1.30)** |  | 0.62 (-0.75, 2.01) |
| Medium-high (>60 to ≤65 dB[A]) |  | -1.06 (-2.42, 0.32) |  | -0.98 (-2.24, 0.31) |  | -0.56 (-1.82, 0.73) |  | 0.11 (-1.32, 1.56) |  | 1.27 (-1.26, 3.85) |
| High (>65 dB[A]) |  | **1.69 (0.23, 3.17)** |  | 0.99 (-0.36, 2.37) |  | 0.50 (-0.83, 1.84) |  | 0.50 (-0.95, 1.98) |  | -0.56 (-3.25, 2.20) |
| **Glycated Haemoglobin, mmol/mol** |  |  |  |  |  |  |  |  |  |  |
| Low (≤55 dB[A]) | 83,613 | reference | 96,253 | reference | 99,974 | reference | 78,288 | reference | 20,762 | reference |
| Low-medium (>55 to ≤60 dB[A]) |  | 0.09 (-0.08, 0.27) |  | 0.12 (-0.03, 0.28) |  | 0.01 (-0.14, 0.15) |  | -0.00 (-0.17, 0.16) |  | 0.01 (-0.31, 0.33) |
| Medium-high (>60 to ≤65 dB[A]) |  | 0.13 (-0.20, 0.47) |  | -0.16 (-0.47, 0.15) |  | -0.14 (-0.44, 0.16) |  | 0.15 (-0.18, 0.48) |  | -0.45 (-1.05, 0.15) |
| High (>65 dB[A]) |  | -0.13 (-0.47, 0.22) |  | **0.44 (0.12, 0.77)** |  | 0.02 (-0.30, 0.33) |  | 0.17 (-0.17, 0.51) |  | -0.21 (-0.80, 0.39) |
| **C- Reactive Protein, mg/L** |  |  |  |  |  |  |  |  |  |  |
| Low (≤55 dB[A]) | 81,946 | reference | 94,836 | reference | 98,431 | reference | 77,506 | reference | 20,539 | reference |
| Low-medium (>55 to ≤60 dB[A]) |  | 0.29 (-0.95, 1.55) |  | -0.52 (-1.67, 0.63) |  | -0.53 (-1.65, 0.60) |  | 0.46 (-0.82, 1.76) |  | **-3.31 (-5.69, -0.86)** |
| Medium-high (>60 to ≤65 dB[A]) |  | -2.57 (-4.92, -0.15) |  | -0.90 (-3.14, 1.41) |  | -1.73 (-3.98, 0.55) |  | 0.99 (-1.58, 3.62) |  | 1.12 (-3.54, 6.00) |
| High (>65 dB[A]) |  | -1.91 (-4.37, 0.62) |  | -0.69 (-3.06, 1.75) |  | -0.57 (-2.91, 1.82) |  | 1.38 (-1.18, 4.02) |  | 0.19 (-4.72, 5.36) |

**Table F6: The association between exposure to road traffic noise and cardiovascular risk factors, stratified by average total household income before tax.**

**Model 3:** Fully adjusted model (Model 2) + adjusted for NO_2_. **Bold** represents significance at p<0.05.
